# Damaged lung gas exchange function of discharged COVID-19 patients detected by hyperpolarized ^129^Xe MRI

**DOI:** 10.1126/sciadv.abc8180

**Published:** 2021-01-01

**Authors:** Haidong Li, Xiuchao Zhao, Yujin Wang, Xin Lou, Shizhen Chen, He Deng, Lei Shi, Junshuai Xie, Dazhong Tang, Jianping Zhao, Louis-S. Bouchard, Liming Xia, Xin Zhou

**Affiliations:** 1 Key Laboratory of Magnetic Resonance in Biological Systems, State Key Laboratory of Magnetic Resonance and Atomic and Molecular Physics, National Center for Magnetic Resonance in Wuhan, Wuhan Institute of Physics and Mathematics, Innovation Academy for Precision Measurement Science and Technology, Chinese Academy of Sciences, Wuhan National Laboratory for Optoelectronics, Wuhan 430071, P. R. China.; 2 Tongji Hospital, Tongji Medical College, Huazhong University of Science and Technology, Wuhan, Hubei 430030, P. R. China.; 3 Department of Radiology, Chinese PLA General Hospital, Beijing 100853, P. R. China.; 4 Jonsson Comprehensive Cancer Center, The Molecular Biology Institute, California NanoSystems Institute, Departments of Chemistry and Biochemistry and of Bioengineering, University of California, Los Angeles, Los Angeles, CA 90095, USA.

## Abstract

The recovery process of COVID-19 patients is unclear. Some recovered patients complain of continued shortness of breath. Vasculopathy has been reported in COVID-19, stressing the importance of probing pulmonary microstructure and function at the alveolar-capillary interface. While computed tomography (CT) detects structural abnormalities, little is known about the impact of disease on lung function. ^129^Xe magnetic resonance imaging (MRI) is a technique uniquely capable of assessing ventilation, microstructure, and gas exchange. Using ^129^Xe MRI, we found that COVID-19 patients show a higher rate of ventilation defects (5.9% versus 3.7%), unchanged microstructure, and longer gas-blood exchange time (43.5 ms versus 32.5 ms) compared with healthy individuals. These findings suggest that regional ventilation and alveolar airspace dimensions are relatively normal around the time of discharge, while gas-blood exchange function is diminished. This study establishes the feasibility of localized lung function measurements in COVID-19 patients and their potential usefulness as a supplement to structural imaging.

## Teaser

Functional imaging of the lungs in COVID-19 patients shows altered gas exchange function.

## INTRODUCTION

In December of 2019, a novel coronavirus disease, COVID-19, caused by the severe acute respiratory syndrome coronavirus 2 (SARS-CoV-2), emerged in Wuhan, China (*1*–*3*). The outbreak of COVID-19 posed a serious threat to global public health (*4*). On 11 March 2020, the World Health Organization (WHO) declared it a pandemic, and more than 7,823,289 cases have been confirmed in more than 200 countries and regions, including 431,541 deaths according to the WHO situation reports (*5*). In China, 78,377 discharged patients were reported to have recovered (*6*), of which about 81% showed mild and moderate symptoms and 14% of cases were considered to be severe. Only 5% of the patients presented acute symptoms (*6*).

COVID-19 is associated with symptoms of hypoxemia and shortness of breath (*7*) as a result of pulmonary dysfunction (*8*, *9*). Autopsy results revealed (*10*) white foam mucus in the trachea, gelatinous mucus attachment in the bronchus, pulmonary edema, and inflammation, indicating that hypoxemia may be related to the damaged pulmonary gas exchange function. In addition, vasculopathy and embolism have also been reported in COVID-19 (*11*), stressing the importance of probing microstructure and function of the lungs at the alveolar-capillary interface. Pulmonary function tests (PFTs) such as FEV_1_ (forced expiratory volume in 1 s)/FVC (forced vital capacity) are used in the clinic to characterize the overall function of the lung. However, PFTs cannot probe vasculature at the alveolar-capillary interface within the lung.

X-ray computed tomography (CT) plays an important role in the diagnosis of lung disease by depicting the pulmonary structure (*12*, *13*). Many COVID-19–confirmed cases exhibit ground-glass opacities (GGOs) and extensive consolidation in the lungs. Although CT images provide high resolution for structural imaging of solid lung lesions, they are unable to evaluate the lung’s gas exchange function. The emerging technique of hyperpolarized (HP) ^129^Xe gas magnetic resonance imaging (MRI) can visualize and quantify both the lung’s microstructure and key functional parameters at the alveolar-capillary interface where gas exchange occurs. Such gas-phase MRI readouts have the potential of detecting the earliest signs of impairment in lung disease (*14*). Moreover, xenon gas MRI offers unique advantages for longitudinal studies, especially those involving children and pregnant women, thanks to the absence of ionizing radiation. Its feasibility and safety have been demonstrated in numerous clinical trials around China, United States, United Kingdom, Canada, and other countries. ^129^Xe MRI has yet to be evaluated in COVID-19 patients, and its potential for characterizing the disease remains unknown.

Here, we present the first ^129^Xe MRI study of discharged COVID-19 patients, aiming at quantitatively evaluating pulmonary gas exchange function and lung microstructure of those patients. We recruited a total of 25 subjects at the start of the outbreak in Wuhan, China—13 discharged COVID-19 patients and 12 healthy volunteers—and performed PFTs and HP xenon gas MRI on all the subjects to derive physiologically relevant parameters such as FEV_1_/FVC, ventilation defects percentage (VDP), exchange time constant (*T*), septal wall thickness (*d*), the ratio of xenon signal from red blood cells and interstitial tissue/plasma (RBC/TP), mean linear intercept (*L*_m_), acinar duct radius (*R*), apparent diffusion coefficient (ADC), and surface-to-volume ratio (SVR). Furthermore, we analyzed the physiological parameters across the two groups and found that, although the lung microstructure appears similar to that of the healthy group, the pulmonary gas exchange function suggests impairment.

## RESULTS

Table 1 summarizes subject data for the 13 discharged COVID-19 patients and 12 healthy subjects as collected upon admission, including demographic, epidemiological, laboratory tests, and clinical findings. Laboratory confirmation of COVID-19 was obtained upon admission to the hospital and subsequently verified by the Wuhan Center for Disease Control and Prevention (CDC). Clinical outcomes were monitored until 5 March 2020. The 13 adult COVID-19 patients were hospitalized in Tongji Hospital (Wuhan, China) before discharge. The admission date ranged from 19 January to 9 February, and the discharge time between 10 February and 5 March. The average age of the discharged patients was 35.8 years, ranging from 25 to 51 years (Table 1). The symptoms upon admission were typically fever, followed by cough and fatigue. Lymphocytopenia occurred in four (30.8%) patients, while six (46.2%) patients had elevated concentrations of high-sensitivity C-reactive protein (>10 mg/liter). Five (38.5%) patients had increased concentrations of alanine aminotransferase and aspartate aminotransferase. In addition, six (46.2%) patients had a normal white cell count, and six (46.2%) patients had a white cell count below the normal range. All patients had been treated in the isolation ward of Tongji Hospital and were deemed sufficiently recovered and discharged with an average hospital stay duration of 23 days.

**Table 1:** Subject demographics and clinical and laboratory findings of discharged patients on admission. ALT, alanine aminotransferase; AST, aspartate aminotransferase; N/A, not applicable.

	**Discharged patients total (*n* = 13)**		**Healthy volunteers total (*n* = 12)**	
**Demographics and clinical characteristics**				
Age, years	35.8 (25.0–51.0)		32.5 (24.0–45.0)	
Sex	Female	Male	Female	Male
	9 (64.3%)	4 (35.7%)	5 (41.7%)	7 (58.3%)
Exposure history	10 (76.9%)		N/A	
Fever (temperature ≥37.3°C)	13 (100.0%)		N/A	
Cough	11 (84.6%)		N/A	
Sputum	4 (30.8%)		N/A	
Myalgia	5 (38.5%)		N/A	
Fatigue	7 (53.8%)		N/A	
Diarrhea	4 (30.8%)		N/A	
Nausea or vomiting	2 (15.4%)		N/A	
Disease severity status	Moderate (100.0%)		N/A	
Time from illness onset to hospital admission, days	7.8 (2–13)		N/A	
**Laboratory findings**				
White blood cell count, ×10^9^/ liter	5.7 (2.7–15.5)		N/A	
<4	6 (46.2%)		N/A	
4 to 10	6 (46.2%)		N/A	
>10	1 (7.6%)		N/A	
Lymphocyte count, ×10^9^/liter	1.2 (0.6–2.5)		N/A	
<0.8	4 (30.8%)		N/A	
Hemoglobin, g/liter	135.4 (111.0–165.0)		N/A	
Platelet count, ×10^9^/liter	242.1 (176–488)		N/A	
ALT, U/liter	36.5 (9.0–84.8)		N/A	
>40	5 (38.5%)		N/A	
AST, U/liter	37.2 (14.0–86.0)		N/A	
>40	5 (38.5%)		N/A	
High-sensitivity C-reactive protein, mg/liter	20.5 (0.8–138.5)		N/A	
>10	6 (46.2%)		N/A	
Interleukin-6, pg/ml	7.2 (1.5–12.9)		N/A	
Confirmatory test done (COVID-19 quantitative reverse transcription polymerase chain reaction)	13 (100.0%)		0 (0%)	

**Fig. 1:**
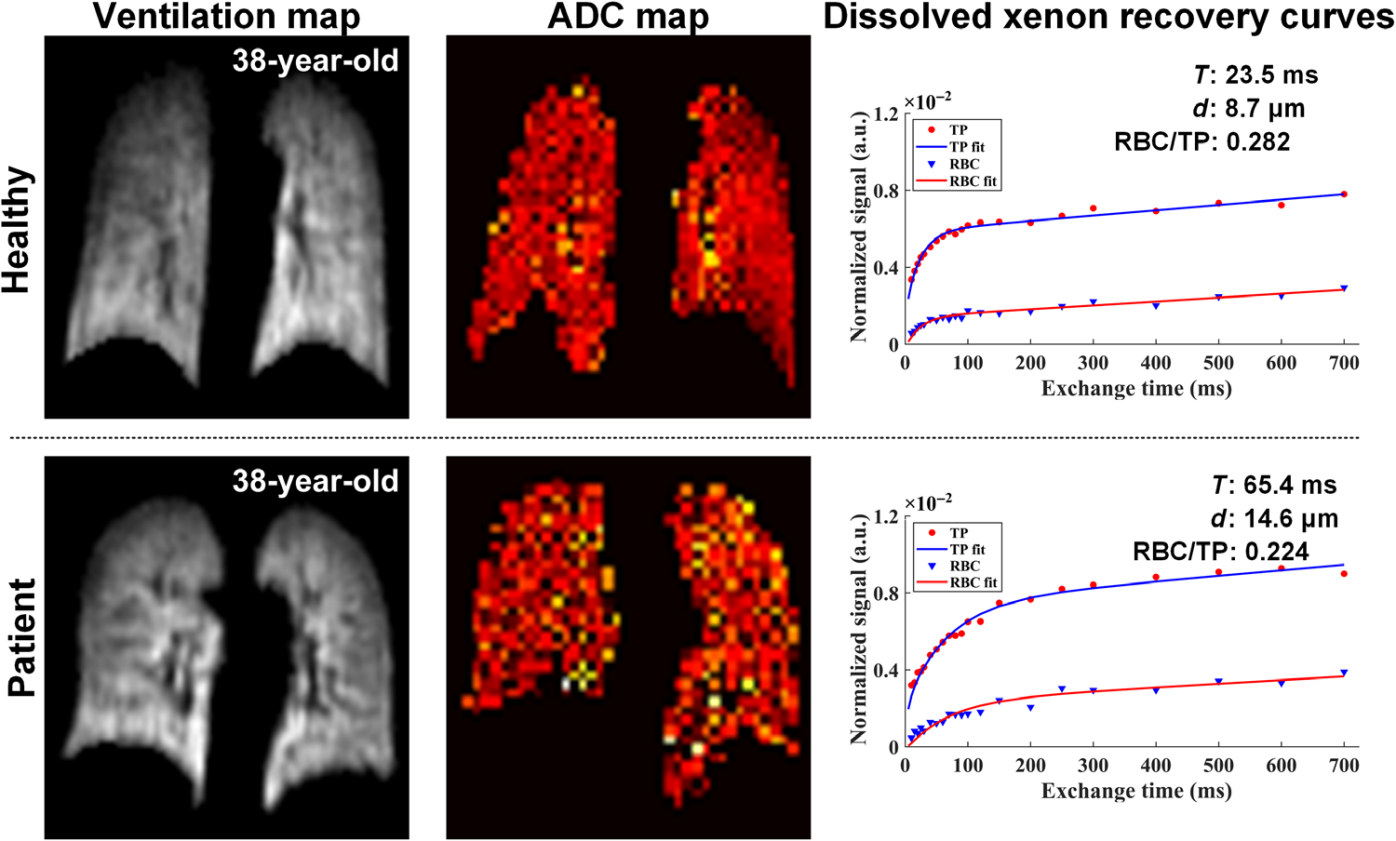
Three typical lung ^129^Xe MRI/MRS datasets collected in this study. Ventilation maps, ADC maps, and dissolved Xe recovery curves are shown for a healthy subject and a discharged COVID-19 patient. Both the healthy and discharged COVID-19 subjects exhibit good ventilation function and microstructure. The VDPs derived from the ventilation maps and microstructural parameters including *L*_m_, *R*, ADC, and SVR derived from diffusion images are comparable in both subjects. However, for the gas exchange function measured by dissolved Xe recovery curves, the discharged COVID-19 patient showed obvious differences compared with healthy subjects. Gas exchange function parameters derived from the xenon recovery curves in healthy subjects versus discharged patients were *T* = 23.5 ms versus 65.4 ms, *d* = 8.7 μm versus 14.6 μm, Hct = 0.260 versus 0.225, and RBC/TP = 0.282 versus 0.224. Note that slice thickness for ADC maps was 30 mm versus 9 mm for ventilation maps. Hence, the two images (ventilation versus ADC) depict slightly different volumes of the lung and do not perfectly match. VDP denotes the ventilation defects percentage; *L*_m_, mean linear intercept; *R*, acinar duct radius; ADC, apparent diffusion coefficient; SVR, surface-to-volume ratio; *T*, exchange time constant; *d*, total septal thickness; Hct, blood hematocrit; RBC/TP, ratio of xenon signal from red blood cells and interstitial tissue/plasma; and a.u., arbitrary units.

Typical ^129^Xe MRI/magnetic resonance spectroscopy (MRS) data from representative discharged COVID-19 patient and healthy volunteer are shown in Fig. 1. VDPs derived from the xenon ventilation map are similar in the healthy subject (subject 6) and the discharged COVID-19 patient (patient 4): 1.5 and 1.6%, respectively. Microstructural parameters derived from the diffusion images, including *L*_m_, *R*, ADC, and SVR, are comparable in both subjects. The gas exchange function parameters derived from the xenon recovery curves show substantial differences in the healthy subject versus the discharged patient: *T* = 23.5 ms versus 65.4 ms, *d* = 8.7 μm versus 14.6 μm, and RBC/TP = 0.282 versus 0.224.

**Fig. 2:**
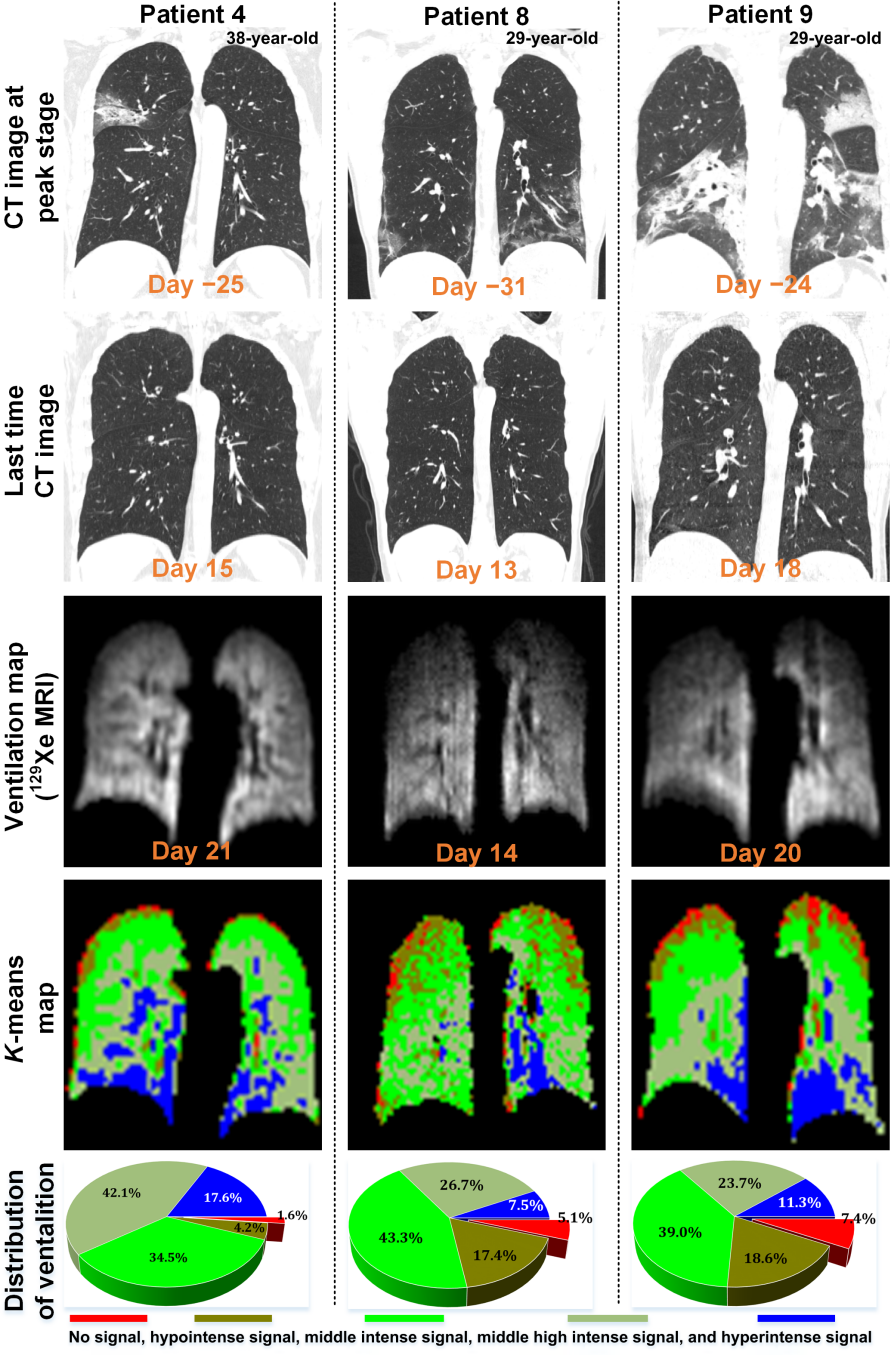
Lung images for three representative discharged patients. From top row to bottom row: Chest CT images at the peak stage of COVID-19, final CT images (upon discharge), corresponding pulmonary ventilation maps from HP ^129^Xe gas MRI, and *k*-means map showing distribution of ventilation intensity. Gas images were segmented through a *k*-means clustering strategy in which voxel intensity values were classified into five clusters ranging from no signal (denoting ventilation defects) to hyperintense signal, therefore generating a gas distribution cluster map, where red color indicates no signal of HP ^129^Xe gas MRI, associated with ventilation defects. The no signal pixels corresponded to the SNR values well within the nuclear MR noise. Brown, green, apple green, and blue color correspond to hypointense, middle intense, middle high intense, and hyperintense gas MRI signals, respectively.

Following antibiotic and antiviral therapies administered in hospital and isolation at home (average interval between the discharge and HP ^129^Xe gas MRI scan: 25 days), representative CT findings in COVID-19 patients (such as GGOs with/without reticular pattern and/or consolidation) at peak stage of COVID-19 pneumonia (Fig. 2) are unexpectedly well absorbed and even almost completely absorbed in some cases (Fig. 2). This would seem to suggest a substantial recovery in the group of discharged patients, at least according to measurements of the lung’s structure via CT.

Xenon gas MRI was then used to perform a more in-depth investigation of lung recovery, as described in Materials and Methods. Maps of lung ventilation distribution are generated and analyzed using a *k*-means clustering strategy in which voxel intensities across entire lungs are classified using one of five labels: no signal (denoting ventilation defects used to derive the VDP metric), hypointense, middle intense, middle high intense, and hyperintense signal. These five clusters are colored in red, brown, green, apple green, and blue, respectively, in Fig. 2. VDP is the percentage of those “ventilation defect” pixels across a given lung that was classified as “no signal.” The distribution of signal intensity from three representative patients is shown in Fig. 2, and the corresponding VDP for these patients are 1.6, 5.1, and 7.4%.

**Fig. 3:**
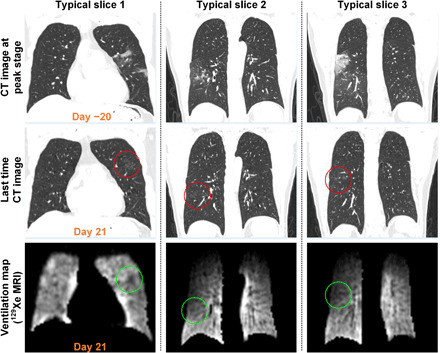
Lung images for subject 2. Serial chest CT images (top row: day −20, three slices; second row: day 21, three slices) and corresponding HP ^129^Xe gas MRI pulmonary ventilation maps (day 21) for one patient who underwent the final CT and gas MRI scans on the same day. In the final follow-up CT images, light GGOs are indicated by red circles. The same GGOs are indicated in the corresponding HP ^129^Xe gas MR images by green circles.

The chest CT scan of a patient acquired at the peak stage of COVID-19 pneumonia reveals multiple GGOs and/or partial consolidation in both the upper and lower lobes of bilateral lungs (Fig. 3). As a result of therapy, those lesions are substantially absorbed in the final scan, although light GGOs with unclear boundaries are still present in Fig. 3 (denoted by red circles). On the same day, the patient underwent an HP ^129^Xe gas MRI scan. The scan reveals signal intensity in the right lung that is lower than that in the left lung. In addition, ventilation in the regions denoted by green circles exhibits hypointense signal that also correlated with structural abnormalities caused by COVID-19 as measured by CT.

Table 2 shows quantitative results for the 13 discharged patients with COVID-19 (cov) and 12 healthy (h) volunteers, including measures of FEV_1_/FVC and %FEV_1_ with PFTs, ventilation function, lung morphometry parameters, and gas-blood exchange function with HP ^129^Xe gas MRI. Five of the 13 (38.5%) the discharged patients have VDP below 5%, and others have VDP within the interval [5%, 10%]. The mean (averaged across each group) VDP was 5.9% for COVID-19 patients and 3.7% for healthy volunteers. Statistical comparisons of VDP via unpaired Student’s *t* test show that COVID-19 patients are statistically different from the healthy volunteers (*P* = 0.0398). The lung morphometry and spirometry parameters in COVID-19 patients, including FEV_1_/FVC, %FEV_1_, *R*, *L*_m_, SVR, and ADC, exhibit no statistically significant differences compared with those of the healthy group. For the discharged patient group, all FEV1/FVC values of patients are consistently above 0.70 (see Table 2). The patient-averaged FEV_1_/FVC value obtained in this study (0.83) is comparable to values reported in the literature for healthy (0.70 in North America, Europe, and China) and subjects with COPD (chronic obstructive pulmonary disease) (<0.70 in North America, Europe, and China) (*15*–*18*). This patient-averaged FEV_1_/FVC value is also similar to the average value that we measured in healthy subjects (0.83). As for the physiological parameters related to gas-blood exchange function measured by HP gas MRI, namely, *T*, *d*, and RBC/TP, all show statistically significant differences (*P* < 0.05) between the two groups (h versus cov). In the discharged patients with COVID-19, *T* and *d* are higher [43.5 ms (cov) versus 32.0 ms (h), and 11.7 μm (cov) versus 10.1 μm (h), respectively], while RBC/TP is reduced [0.279 (cov) versus 0.330 (h)] when compared with that in healthy volunteers. Quantitative results for each healthy subject are summarized in table S1.

**Table 2:** Quantitative results of PFTs and HP ^129^Xe gas MRI for discharged COVID-19 patients. Note that *R*, *L*_m_, SVR, and ADC of the patient values cited are means ± SD over all pixels in the lung. Duration of hospitalization in the second row denotes hospitalization days. Last time in the fourth row denotes days from discharge, and scan time in the 10th row denotes days from discharge. SS denotes stripe shadow, ACA denotes representative findings in COVID-19 patients that were almost completely absorbed, GS denotes grid shadow, PS denotes patchy shadow, SPS denotes salt and pepper signs, and %FEV_1_ denotes PFTs expressed as a percentage of a predicted value. The individual patient data for healthy subjects are found in table S1.

**Subject no.**	**1**	**2**	**3**	**4**	**5**	**6**	**7**	**8**	**9**	**10**	**11**	**12**	**13**	**Mean**	**Healthy**	***P*** **value**
Duration of hospitalization (days)	11	24	13	29	29	13	18	36	28	18	22	21	28	23	–	–
**Chest CT scan**																
Last time	Day 37	Day 21	Day 20	Day 15	Day 13	Day 29	Day 11	Day 13	Day 18	Day −7	Day −1	Day 12	Day 15	–	–	–
Characteristics	GGOs	GGOs + SS	ACA	ACA	GGOs + SS + GS	ACA	GGOs + PS	GGOs + PS	GGOs + PS	GGOs + SPS	ACA	ACA	ACA	–	–	–
**PFTs**																
FEV_1_/FVC	0.82	0.84	0.87	0.83	0.80	0.81	0.81	0.87	0.83	0.88	0.81	0.82	0.77	0.83	0.83	0.7712
%FEV_1_ (%)	139	143	154	155	133	150	156	199	149	150	120	151	98	146	141	0.6386
**HP ^129^Xe gas MRI**																
Scan time	Day 34	Day 21	Day 27	Day 21	Day 20	Day 27	Day 26	Day 14	Day 20	Day 29	Day 20	Day 32	Day 29	–	–	–
**Gas-gas exchange function**																
VDP (%)	9.5	9.9	2.3	1.6	9.9	5.6	8.4	5.1	7.4	4.9	2.9	5.5	4.0	5.9	3.7	0.0398
**Gas-blood exchange function**																
*T* (ms)	37.7	50.0	28.3	65.4	63.3	–	59.9	20.3	46.4	39.6	52.6	30.2	28.1	43.5	32.0	0.0380
*d* (μm)	11.1	12.8	9.6	14.6	14.4	–	14.0	8.1	12.3	11.4	13.1	9.9	9.6	11.7	10.1	0.0435
Hct	0.193	0.212	0.187	0.225	0.180	–	0.199	0.263	0.331	0.189	0.220	0.134	0.204	0.211	0.242	0.1124
RBC/TP	0.279	0.308	0.219	0.224	0.272	–	0.235	0.323	0.302	0.262	0.340	0.337	0.252	0.279	0.330	0.0410
**Lung morphometry parameters**																
*R* (μm)	327 ± 66	337 ± 77	335 ± 72	345 ± 80	327 ± 69	331 ± 67	352 ± 80	351 ± 82	351 ± 82	327 ± 67	355 ± 74	347 ± 84	328 ± 69	339	337	0.6751
*L*_m_ (μm)	188 ± 54	221 ± 81	199 ± 63	222 ± 71	206 ± 72	189 ± 56	218 ± 61	222 ± 69	220 ± 70	202 ± 67	215 ± 50	222 ± 78	200 ± 62	210	203	0.3470
SVR (cm^−1^)	228 ± 57	202 ± 63	216 ± 55	196 ± 54	214 ± 62	227 ± 56	197 ± 52	195 ± 53	198 ± 56	215 ± 58	195 ± 39	199 ± 59	216 ± 55	208	214	0.3572
ADC (cm^2^/s)	0.032 ± 0.012	0.033 ± 0.019	0.032 ± 0.014	0.035 ± 0.016	0.027 ± 0.023	0.030 ± 0.013	0.036 ± 0.011	0.037 ± 0.013	0.036 ± 0.012	0.029 ± 0.019	0.037 ± 0.008	0.033 ± 0.022	0.033 ± 0.013	0.033	0.032	0.5688

## DISCUSSION

As an emerging technique for quantifying pulmonary function in pulmonary diseases, the feasibility of HP xenon gas MRI in evaluating gas exchange function and microstructural parameters of the lung has been demonstrated in multiple clinical studies (*18*–*23*). However, none of them have dealt with COVID-19 patients. By using HP xenon gas MRI, hypoxemia in a COVID-19 patient caused by poor ventilation or gas exchange or both can be determined. Here, the effects of COVID-19 on the lungs of discharged patients have been evaluated. The average VDP of the discharged patients was 5.9%, which is significantly larger than that in healthy subjects (3.7%). We note that all recovered patients had a VDP of less than 10%. The statistical differences between the discharged and healthy subjects (*P* = 0.0398) meant that the lesions after absorption (e.g., light GGOs) may have an effect on ventilation function. This is evident in Fig. 3, where the location of lesions (red circles) exhibited low signal intensity in the ventilation maps. Such a detailed assessment of lung function is not currently possible by PFT.

We also evaluate pulmonary morphological parameters using diffusion-weighted MRI (DW-MRI). We recorded microstructural parameters of the pulmonary acinus, which are the largest lung units involved in pulmonary gas exchange, located at the distal end of the terminal bronchioles. The measured ADC, *R*, *L*_m_, and SVR in discharged patients have values similar to those of healthy subjects. Morphological parameters are also found to be within normal ranges in some discharged patients that had GGO(s) visible in their final CT scan. HP ^129^Xe gas MRI was used to image the alveolar airspace. The pulmonary morphological parameters were derived on the basis of the diffusion properties of xenon gas in the alveoli. Our results indicate that the alveolar microstructure for gas diffusion did not appear affected in most COVID-19 patients after treatment (recalling that our COVID-19 patients had moderate condition).

One of the sequelae of SARS is pulmonary fibrosis (*24*). The gas exchange function parameters *T*, *d*, and RBC/TP measured with HP ^129^Xe MR were found significantly different between healthy subjects and patients with interstitial lung diseases (ILDs) such as pulmonary fibrosis (*23*, *25*). Recent studies revealed that the pathological features of COVID-19 are similar to those of SARS; for example, in SARS, interstitial mononuclear inflammatory infiltrates and fibroblastic proliferation of alveolar septum are also found in the lung (*10*, *26*, *27*). In our study, we found that *T* and *d* were 35.9 and 15.8% higher, respectively, whereas RBC/TP was 17.8% lower in discharged patients (relative to healthy subjects). Those observations are similar to the trends observed in patients with ILD (*23*, *25*). On the basis of a simple comparison of two time points in four discharged patients (fig. S2 and table S2), we attribute them to the interstitial thickening and perfusion deficits caused by inflammation and possible fibrosis (*10*) during the patients’ recovery stage, which would be expected to increase upon xenon uptake and slow down the transfer of xenon from alveoli to RBCs. The relatively preserved lung function and lack of microstructural damage in discharged COVID-19 patients may be attributed to the relatively young age of the discharged patients (average age, 35.8 years). Our results suggest that the pulmonary function parameters obtained using HP gas MRI are more sensitive for the evaluation of pulmonary injuries caused by COVID-19 than PFT and CT combined, in particular the parameters related to gas exchange function. (The measured *d* in this study is a “functional” septal wall thickness, not the morphological one.)

This study shows that HP ^129^Xe MRI offers a unique advantage in evaluating pulmonary function damage caused by COVID-19. X-ray CT, which has played a critical role in the diagnosis and monitoring of therapy for the COVID-19 global health emergency, features high accuracy and short scan times (*28*). According to patient discharge criteria in China (*29*), lesions in representative CT lung scans (e.g., GGOs and/or consolidation in Fig. 2) for discharged patients should be substantially absorbed. After more than 14 days of isolation, those structural abnormalities are further (and in a case, almost completely) absorbed. Nevertheless, there are large differences among the three patients with regard to both ventilation function (e.g., VDP of 1.6, 5.1, and 7.4%) and gas-blood exchange function (e.g., *T*, *d*, and RBC/TP) of the lung, yet little difference in the microstructural parameters. These findings indicate that compared with lung structure, gas-blood exchange function of the lung may require a longer time to recover, and the technique of HP ^129^Xe gas MRI here is critical in providing this type of assessment.

HP gas MRI scans were typically carried out a few days following the corresponding final CT scans, except for subject 2, who underwent both MRI and CT scans on the same day. For subject 2, multiple GGOs in the upper and lower lobes of bilateral lungs are significantly enlarged (Fig. 3), relative to the initial CT images, which meant that the absorption of structural abnormalities would be expected to require more time because of the presence of multiple light GGOs in the left upper and lower lobes of bilateral lungs with unclear boundaries (Fig. 3). HP ^129^Xe gas MRI reveals that the lesions in Fig. 3 had low signal intensity. By combining HP gas MRI with the corresponding CT images, the physician is provided with a comprehensive picture of the lung state based not only on microstructural information (e.g., size, location, and morphological features of lesions) but also on gas exchange function (e.g., ventilation, gas-blood exchange, and microstructure parameters). Xenon images provide unique information about the lung function that is absent from structural images. The correlation between structure and function remains unclear: Patients that appear to have recovered based on CT scans may still have impaired lung function according to the ventilation maps. In these cases, xenon MRI is able to quantify the extent and nature of the functional impairment and is expected to be helpful for prognosis and monitoring of COVID-19 disease progression (e.g., the enlargement or absorption of GGOs and/or consolidation and the recovery of lung structure and function).

Our research can be further expanded, since xenon MRI scans only performed on the participants at a single point in time (upon discharge). It would be interesting to perform longitudinal studies on those patients to assess recovery over the long term. This study only probed gas exchange on the whole lung. To better understand the local functional impairment, regional thickening of the interstitial tissue barrier and regional defects in RBCs should be the subject of future studies (*30*–*32*). Last, we used a flexible vest radio frequency (RF) coil to collect the MRI data. Such coils exhibit a moderate amount of B_1_ inhomogeneity, namely, signal loss near the edges of the coil (such as at the apex of the lung). To evaluate the ventilation defects quantitatively within a specific patient group, coils with more homogeneous B_1_ distribution and an improved bias field correction algorithm may be helpful (*33*–*35*).

In summary, we evaluated the pulmonary gas exchange function and microstructural parameters in 13 COVID-19 discharged patients with moderate symptoms using the imaging technique HP ^129^Xe gas MRI. We found statistically significant differences in *T*, *d*, VDP, and RBC/TP between the cohorts, highlighting the power of xenon MRI in evaluating lung injury caused by COVID-19. HP ^129^Xe gas MRI could be a powerful supplement to chest CT in evaluating the lung’s gas exchange function in COVID-19 patients and may be useful in longitudinal follow-ups of patients during progress of disease and subsequent recovery of lung function.

## MATERIALS AND METHODS

According to the Chinese Clinical Guidance for COVID-19 Pneumonia Diagnosis and Treatment (seventh edition) issued by the National Health Commission of China (*29*), clinical classifications are divided into mild, moderate, severe, and critically severe symptoms. According to the guide’s discharge standard, patients that meet the following conditions can be discharged: (i) The body temperature returns to normal for more than three consecutive days; (ii) significant improvement of the respiratory symptoms; (iii) pulmonary CT images showing significant improvement of the acute exudative lesions; (iv) and negative nucleic acid test results of sputum, nasopharynx swab, and other respiratory samples for two consecutive tests (with sampling time at least 1 day apart). A total of 13 COVID-19 pneumonia discharged patients (4 men and 9 women, average age is 35.8 years) with moderate symptoms were recruited for our study. All patients had been laboratory confirmed for COVID-19 when first admitted to the hospital and subsequently verified by the Wuhan CDC. They have no history of lung disease and smoking, and lung health was also confirmed by chest x-ray examination, which was performed in the annual physical examination (year 2019). Clinical records, laboratory results, and chest CT scans were retrospectively reviewed for these discharged patients, who were admitted to Tongji Hospital, Tongji Medical College, Huazhong University of Science and Technology, Wuhan, China. We also recruited 12 healthy volunteers with an average age of 32.5 years (range, 24 to 40 years) as the control group. Our study was approved by the Ethics Commission of Tongji Medical College, Huazhong University of Science and Technology, and all subjects signed the informed consent forms.

Final CT scans were obtained with patients in the supine position (11 patients underwent scanning after discharge, and two patients underwent scanning shortly before discharge) using one of the three CT systems: LightSpeed 16 or volume CT, GE Healthcare, Wisconsin, USA (9/13); Somatom Definition AS+, Siemens, Germany (2/13); and uCT 780, United Imaging, China (2/13). The following parameters were used: tube voltage = 100 to 120 kilovolt peak, matrix = 512 × 512, slice thickness = 1.00 to 1.25 mm, and field of view (FOV) = 350 mm × 350 mm. The tube current was regulated through an automatic exposure modulation system, and the single collimation width of reconstructed images was 0.50 to 1.25 mm.

All MRI scans were performed using a 3.0-T multinuclear whole-body MRI Scanner [uMR780(Xe), Wuhan, China] with a horizontal magnet using a transmit-receive vest RF coil (35.45 MHz for ^129^Xe). HP ^129^Xe was produced with the spin-exchange optical pumping technique using a commercial polarizer system (verImagin Healthcare, Wuhan, China), with a freeze-out accumulation procedure in a cold finger. A gas mixture of 2% enriched xenon (86% ^129^Xe isotope), 88% ^4^He, and 10% N_2_ was used in this study (*36*). After the xenon accumulation process, HP gas was thawed using hot water and extracted into a 1-liter Tedlar bag, which was purged to approximately 9 Pa with a vacuum pump. The available spin polarization of ^129^Xe gas in the Tedlar bag was approximately 25%. Before the magnetic resonance (MR) experiments, the subject inhaled 1 liter of gas mixture (5% xenon + 95% N_2_) from the functional residual capacity (FRC) to calibrate the flip angle. Afterward, the subject inhaled 1 liter of gas mixture (40% xenon + 60% N_2_) from the FRC for measuring pulmonary ventilation function, gas exchange function, and microstructure parameters, respectively. For each patient, a total of four bags of xenon gas were used. The patients were kept in the MRI scanner for less than 15 min; for each xenon MRI scan, the duration was less than 15 s.

**Fig. 4:**
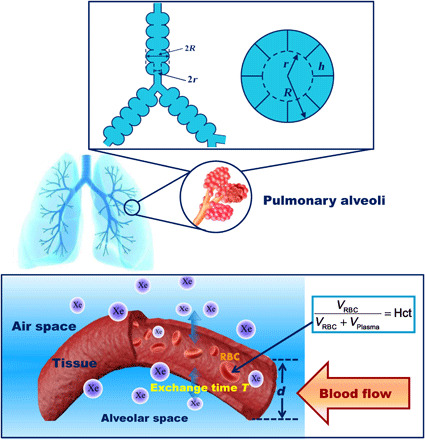
Sketch illustrating pulmonary microstructure parameters (top) and gas-blood exchange function parameters (bottom) of the lung as provided by HP ^129^Xe gas MRI. The top schematic shows the two generations of lung respiratory airways of the Weibel lung model: The main morphometric parameters include external radius (*R*), internal radius (*r*), and depth of alveolar sleeve (*h*). The bottom illustration shows the diagram of the gas-blood exchange region of the alveoli. The total septal thickness is assumed to be *d*. *T* is the xenon gas exchange time constant in the lung. Hct is the hematocrit of the lung.

Pulmonary ventilation function was assessed using HP ^129^Xe gas MRI. The MRI scans were performed on each subject using a three-dimensional balanced steady-state free precession sequence with FOV = (38 cm)^2^, matrix = 96 × 84, slice number = 20, slice thickness = 9 mm, bandwidth = 800 Hz per pixel, flip angle = 12° to 14°, and repetition time/echo time = 5.6/2.65 ms. Proton images of the thoracic cavity for matching were also acquired using the ^1^H body coil after the ^129^Xe MRI scan during the same breath-hold. Proton MRI scans were acquired using a fast-spoiled gradient-echo sequence with FOV = (38 cm)^2^, matrix = 96 × 48 (partial echo = 82%), number of slices = 20, slice thickness = 9 mm, bandwidth = 800 Hz per pixel, flip angle = 20°, and repetition time/echo time = 4.5/2.2 ms. All ^129^Xe gas and proton images were reconstructed to a 96 × 96 matrix for purposes of calculating the ventilation defects. In this study, we did not perform a retrospective bias field correction, which has been used by other groups for correcting B_1_ inhomogeneity (*33*–*35*), because these corrections often inadvertently remove intensity nonuniformity caused by physiological changes. The technique of HP ^129^Xe chemical shift saturation recovery (CSSR) was used to obtain the gas-blood exchange functional parameters of the lung by the model of xenon exchange (MOXE) using a dissolved-phase ^129^Xe diffusion coefficient of 3.3 × 10^−6^ cm^2^ s^−1^ (*16*). For the CSSR data collection, 21 exchange time points ranging from 10 to 700 ms were used, and the spectra were acquired using a 90° RF pulse with a bandwidth of 50 Hz per points and 1024 sampling points. The bottom part of Fig. 4 shows the physiological parameters, including *T*, *d*, and blood hematocrit (Hct). The ratio of xenon signal from RBCs and xenon signal from interstitial TP at an exchange time of 100 ms was calculated (RBC/TP) (*17*, *25*). HP ^129^Xe DW-MRI images were used to derive the pulmonary microstructure parameters. DW-MRI scans were acquired for four *b* values (0, 10, 20, and 30 s/cm^2^) using interleaved sampling and acquisition parameters: FOV = (38 cm)^2^, matrix = 64 × 32, slice number = 4, slice thickness = 30 mm, bandwidth = 600 Hz per pixel, flip angle = 8° to 11°, and repetition time/echo time = 15.5/12.5 ms. As shown in the top part of Fig. 4, the pulmonary morphometric parameters, including *R*, *L*_m_, and SVR, were extracted by fitting the DW-MRI data to the anisotropic diffusion model (*37*) of the ^129^Xe diffusion pixel by pixel using a nonlinear least squares algorithm after reconstructing the images to 64 × 64 matrix (*18*). PFTs were performed on the same day as the MRI scans using a handheld spirometer (sp-1, Schiller AG, Switzerland). MRI-derived microstructural parameters *R*, *L*_m_, SVR, and ADC were computed as maps (i.e., on a pixel-by-pixel basis); however, the values reported in this article are volume averaged over the patient’s lungs, and the error bars cited are SDs over the same region.

## Supplementary Material

http://advances.sciencemag.org/cgi/content/full/sciadv.abc8180/DC1

Adobe PDF - abc8180_SM.pdf

Damaged lung gas exchange function of discharged COVID-19 patients detected by hyperpolarized 129Xe MRI

## SUPPLEMENTARY MATERIALS

Supplementary material for this article is available at http://advances.sciencemag.org/cgi/content/full/sciadv.abc8180/DC1

## Appendix

View/request a protocol for this paper from *Bio-protocol*.
