# Supplementary material for: Damaged lung gas exchange function of discharged COVID-19 patients detected by hyperpolarized 129Xe MRI
Source: Sci Adv. 2021 Jan 1;7(1):eabc8180. doi: 10.1126/sciadv.abc8180 (PMC7775756; doi:10.1126/sciadv.abc8180)
Supplement: http://advances.sciencemag.org/cgi/content/full/sciadv.abc8180/DC1 [file abc8180_index.html]

Science Advances | Science AdvancesAAASSearchScience AdvancesMenu

## Supplementary Materials

# Damaged lung gas-exchange function of discharged COVID-19 patients detected by hyperpolarized 129Xe MRI

Haidong Li, Xiuchao Zhao, Yujin Wang, Xin Lou, Shizhen Chen, He Deng, Lei Shi, Junshuai Xie, Dazhong Tang, Jianping Zhao, Louis-S. Bouchard, Liming Xia, Xin Zhou

Download Supplement

**This PDF file includes:**

- Fig. S1
- Tables S1 to S2

**Files in this Data Supplement:**

- Adobe PDF - abc8180\_SM.pdf
