# Supplementary material for: Damaged lung gas exchange function of discharged COVID-19 patients detected by hyperpolarized 129Xe MRI
Source: Sci Adv. 2021 Jan 1;7(1):eabc8180. doi: 10.1126/sciadv.abc8180 (PMC7775756; doi:10.1126/sciadv.abc8180)
Supplement: http://advances.sciencemag.org/cgi/content/full/sciadv.abc8180/DC1 [file abc8180_1.pdf]

Science Advances
AAAS

advances.sciencemag.org/cgi/content/full/sciadv.abc8180/DC1

Supplementary Materials for

Damaged lung gas-exchange function of discharged COVID-19 patients detected by hyperpolarized ^{129}Xe MRI

Haidong Li, Xiuchao Zhao, Yujin Wang, Xin Lou, Shizhen Chen, He Deng, Lei Shi, Junshuai Xie, Dazhong Tang, Jianping Zhao, Louis-S. Bouchard, Liming Xia*, Xin Zhou*

*Corresponding author. Email: xinzhou@wipm.ac.cn (X. Z.) and lmxia@tjh.tjmu.edu.cn (L. X.)

Published 20 November 2020, *Sci. Adv.* **6**, eabc8180 (2020)
DOI: 10.1126/sciadv.abc8180

This PDF file includes:

Fig. S1
Tables S1 to S2

Table S1. Quantitative results of pulmonary function tests and hyperpolarized ^{129}Xe gas MRI for healthy volunteers.

Subject No.	1	2	3	4	5	6	7	8	9	10	11	12	Mean
Pulmonary function tests													
FEV_1/FVC (%)	0.856	0.811	0.753	0.849	0.839	0.913	0.816	0.860	0.844	0.899	0.822	0.732	0.833
%FEV_1 (%)	114	126	107	139	147	148	159	158	128	147	192	132	141
Hyperpolarized ^{129}Xe gas MRI													
Ventilation function													
VDP (%)	8.3	4.5	2.3	1.9	5.3	1.5	0.6	3.6	4.6	3.7	2.3	5.9	3.7
Gas-blood exchange function													
T (ms)	47.2	24.2	19.0	30.4	34.4	23.5	42.2	41.1	22.3	40.2	24.8	35.1	32.0
d (μm)	12.4	8.9	7.9	9.9	10.6	8.7	11.7	11.6	8.5	11.4	9.0	10.7	10.1
Hct	0.229	0.232	0.343	0.259	0.174	0.260	0.240	0.254	0.211	0.221	0.227	0.249	0.242
RBC/TP	0.365	0.284	0.354	0.307	0.245	0.282	0.253	0.481	0.285	0.371	0.344	0.387	0.330
Lung morphometry parameters													
R (μm)	349±78	349±81	330±70	323±66	327±69	347±77	332±66	317±65	365±86	352±79	331±67	325±69	337
L_m (μm)	216±62	215±65	189±55	183±56	187±56	213±59	186±45	183±62	252±74	215±61	189±53	206±76	203
SVR (cm^{-1})	199±50	201±53	226±55	236±59	229±55	201±50	226±51	237±62	171±45	198±47	226±52	216±63	214
ADC (cm^2/s)	0.036±0.012	0.036±0.012	0.030±0.014	0.027±0.017	0.0298±0.013	0.036±0.011	0.031±0.010	0.025±0.021	0.042±0.010	0.036±0.011	0.031±0.012	0.028±0.020	0.032

Note: FEV_1 denotes the forced expiratory volume in 1 s, FVC denotes the forced vital capacity, %FEV_1 denotes pulmonary function tests expressed as percentage of predicted value, VDP denotes the ventilation defects percentage, ADC denotes the apparent diffusion coefficient, R denotes acinar duct radius, L_m denotes mean linear intercept, SVR denotes surface-to-volume ratio, T denotes the exchange time constant, d denotes the total septal thickness, Hct denotes the blood hematocrit, and RBC/TP denotes the ratio of xenon signal from red blood cells and interstitial tissue/plasma, respectively. For individual patients, R, L_m, SVR, ADC values cited are mean ± standard deviation obtained over all pixels covering the lung region.

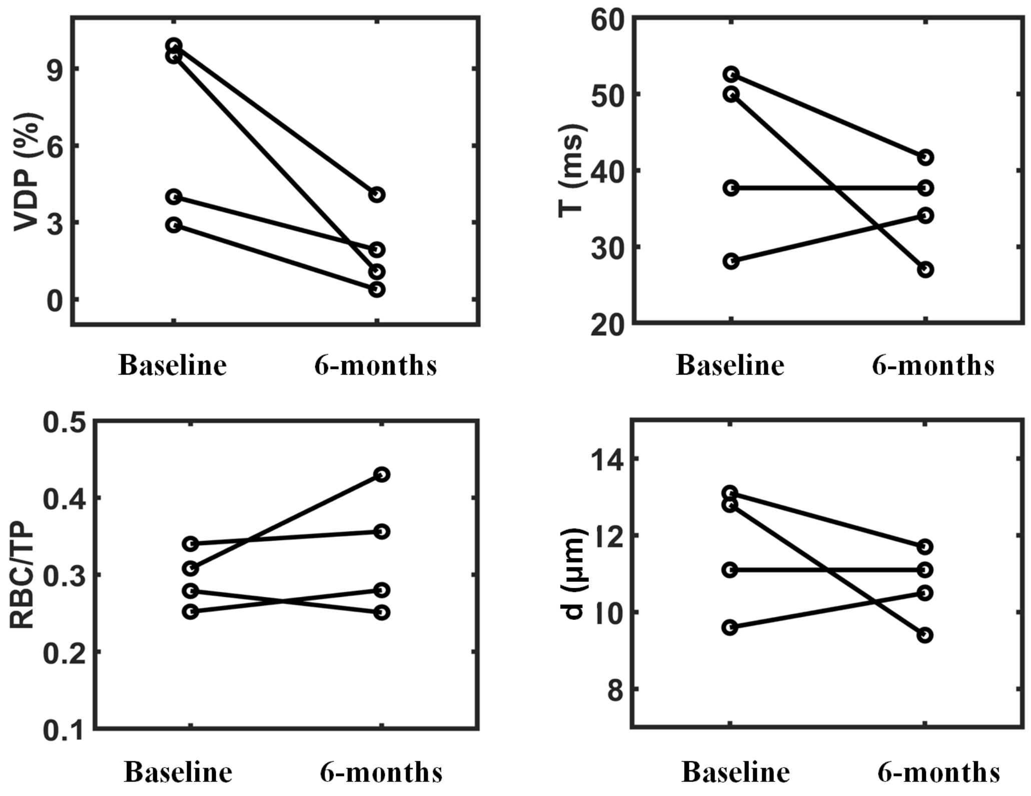

Fig. S1. Longitudinal change of VDP, T, d and RBC/TP in four discharged patients. The measured ventilation function and gas exchange function parameters changed after six months following the first post-discharge ^{129}Xe MRI scan. VDP decreased in all four discharged patients, which means that the ventilation function improved after a recovery period of six months. For the gas exchange function, the results vary from person to person. The measured RBC/TP increased in three discharged patients, which means that the lung function improved, except for one patient where it deteriorated. T and d decreased in two discharged patients, which means that the gas-exchange function improved, stayed almost unchanged in one patient, but increased in another patient. Baseline denotes the first ^{129}Xe MRI scan time point following discharge.

Table S2. The measured VDP, T, d and RBC/TP at baseline and six months following the first ^{129}Xe MRI scan after discharge.

Subject No.		VDP (%)	*T* (ms)	*d* (μm)	RBC/TP
**1**	Baseline	9.5	37.7	11.1	0.279
	6-months	1.1	37.7	11.1	0.251
**2**	Baseline	9.9	50.0	12.8	0.308
	6-months	4.1	27.0	9.4	0.430
**11**	Baseline	2.9	52.6	13.1	0.340
	6-months	0.4	41.7	11.7	0.356
**13**	Baseline	4.0	28.1	9.6	0.252
	6-months	1.9	34.1	10.5	0.280

Note: Baseline denotes the first ^{129}Xe MRI scan time after discharge and 6-months denotes the total timeframe of the two examinations is about six months (196 ± 2 days).
